# The Effectiveness of Endoscopic Ultrasonography and Computed Tomography in the Differentiation of Pancreatic Cystic Neoplasms: A Single-Center Experience

**DOI:** 10.5152/tjg.2023.23492

**Published:** 2024-12-01

**Authors:** Bengi Öztürk, Koray Ceyhan, Mehmet Bektaş

**Affiliations:** 1 Department of Internal Medicine, Ankara University Faculty of Medicine, Ankara, Türkiye; 2 Department of Cytopathology, Ankara University Faculty of Medicine, Ankara, Türkiye; 3 Department of Gastroenterology, Ankara University Faculty of Medicine, Ankara, Türkiye

**Keywords:** Endoscopic ultrasonography, pancreatic cysts, fine needle aspiration

## Abstract

**Background/Aims:**

Radiological imaging advancements have led to a rise in pancreatic cyst diagnoses. Apart from imaging modalities, endoscopic ultrasonography (EUS) is an important method in the diagnosis of pancreatic cysts. The aim of this study is to determine the clinical, laboratory, biochemical, radiological, and endosonographic features of pancreatic cysts and to assess the effectiveness of EUS and computed tomography (CT) in differentiating between neoplastic and nonneoplastic cysts.

**Materials and Methods:**

Patients with pancreatic cysts diagnosed by CT, who underwent EUS at the EUS Laboratory of Ankara University Faculty of Medicine, Gastroenterology Department, between 2010 and 2015, were retrospectively evaluated. Size, location, number, and morphological features were compared. Samples for cytology and biochemical tests were obtained through EUS-guided fine needle aspiration (EUS-FNA).

**Results:**

The study group included 212 patients. Among them, 125 (59%) patients underwent EUS-FNA. Of these, 63 (52%) were neoplastic, 29 (24%) were nonneoplastic, and 29 (24%) lacked subgroup analysis. The sensitivity of CT in differentiating between neoplastic and nonneoplastic cysts was 82.1% [CI, 68.2%-91.9%], with a specificity of 61.1% (CI, 38.2%-81%) and diagnostic accuracy of 75.4%. Regarding EUS, the sensitivity was 96.7% (CI, 90.2%-99.4%), with a specificity of 45.8% (CI, 27.1%-65.4%) and diagnostic accuracy of 82.3%.

**Conclusion:**

Endoscopic ultrasonography demonstrated enhanced sensitivity compared with CT in differentiating neoplastic from nonneoplastic pancreatic cysts. Although no statistical significance was found, this result can be considered clinically remarkable. In addition, EUS displayed distinct advantages in visualizing specific morphological features, emphasizing its potential as a valuable diagnostic tool in assessing pancreatic cystic neoplasms.

**Main Points**

- The clinical, laboratory, biochemical, radiological, and endosonographic features of pancreatic cysts were determined, and the effectiveness of endoscopic ultrasonography (EUS) and computed tomography (CT) in differentiating between neoplastic and nonneoplastic cysts was assessed.
- The sensitivity of EUS in differentiating between neoplastic and nonneoplastic cysts was higher than that of CT.
- Endoscopic ultrasonography showed septation, mural nodule, connection to the pancreatic duct, thickened cyst wall, and dilation of the main pancreatic duct more clearly than CT.

## Introduction

Pancreatic cysts are increasingly diagnosed due to the widespread use of radiological imaging techniques at present. Pancreatic cysts have been detected in 2% of patients who underwent computed tomography (CT) and magnetic resonance imaging (MRI) for other indications, and this rate increases with age.^1^ Although it was previously thought that approximately 15% of pancreatic cysts were neoplastic, 85% were pseudocysts (PCs); at present, it is emphasized that more than half of pancreatic cysts have a neoplastic character.^2^ Pancreatic cystic neoplasms (PCNs) include mucinous cystic lesions (MCLs) [mucinous cystic neoplasms (MCNs) and intraductal papillary mucinous neoplasms (IPMNs)], serous cystic neoplasms (SCNs), and solid pseudopapillary neoplasms (SPNs). The most important diagnostic methods for pancreatic cysts are as follows: sectional imaging techniques such as CT and MRI, magnetic resonance cholangiopancreatography (MRCP), endoscopic retrograde cholangiopancreatography, endoscopic ultrasonography (EUS), EUS-guided fine needle aspiration (EUS-FNA) for cyst fluid analysis (amylase, tumor markers, DNA analysis, and cytological evaluation), and cyst wall brushing.^3^

A cyst size larger than 3 cm, presence of a thickened cyst wall, mural nodule, main pancreatic duct dilation (caliber ≥5-9 mm), lymph node, and enhancing solid component on radiological imaging were all defined as worrisome features.^4,5^ According to EUS, malignant morphological features of the cysts are defined as similar to the radiological imaging findings, such as the presence of a thickened cyst wall, thick septation, mural nodule, and main duct dilation.^6^ Although CT is commonly used in the diagnosis of pancreatic cysts, it is inadequate in the differential diagnosis of malignant lesions from benign ones. Diagnostic accuracy of CT varies between 20% and 95%.^7,8^ In various studies, very different results ranging from 51% to 92%-96% were reported for diagnostic accuracy of EUS in differentiating between malignant and benign cysts in PCN.^9^ Endoscopic ultrasonography is also inadequate for differentiating between malignant and benign pancreatic cysts. The rate of diagnostic accuracy in distinguishing malignant from benign cysts increases with the addition of FNA to EUS.^10,11^

The aim of our study was to determine the clinical, laboratory, radiological, and endoscopic ultrasonographic characteristics of pancreatic cysts and to assess the effectiveness of EUS and CT for differentiating between neoplastic and nonneoplastic pancreatic cysts (NNPCs) diagnosed cytopathologically.

## Materials and Methods

### Patients

Patients diagnosed with pancreatic cysts by CT and who underwent EUS at the EUS Laboratory of Ankara University Faculty of Medicine, Gastroenterology Department between 2010 and 2015 were retrospectively evaluated. Patients diagnosed with pancreatic cysts by imaging techniques other than CT, such as MRI, MRCP, and abdomen ultrasonography, were not included in the study. Informed consent was not required because of the retrospective design of the study. This study was approved by Ethics Committee of Ankara University Faculty of Medicine (date: April 14, 2014; decision number: 06-258-14).

In our study, pancreatic cysts were categorized into neoplastic cysts and nonneoplastic cysts as follows: SCNs, MCNs, IPMNs, and SPNs were classified as neoplastic cysts, whereas true cysts and PCs were defined as nonneoplastic cysts.

Approval for the study was obtained from Ankara University Faculty of Medicine Local Ethics Committee (Date: April 14, 2014; Decision number:06-258-14).

#### Computed Tomography

For conventional abdominal CT scanning, all patients were orally administered 2 L of 2% contrast solution for 2 hours. In addition, 120 mL of low molecular weight iodine-containing contrast agent was administered at a dose of 300 mg/mL (Omnipaque) 3 mL/s. A contrast agent was administered via the intravenous (IV) route, and upper abdominal CT sections were obtained. The pancreatic region was screened with 3 mm sections. The pancreas and abdomen were evaluated during the early arterial phase. In the second stage, 70-75 seconds after IV injection, the pancreas was evaluated at the portal and hepatic phases.

#### Endoscopic Ultrasonography

Endoscopic ultrasonography was performed by a single experienced endoscopist at Ankara University Faculty of Medicine, Department of Gastroenterology. Endoscopic ultrasonography was performed after overnight fasting. Before EUS, sedation was performed with midazolam or midazolam–propofol–fentanyl. For the procedure, Fujinon 4400SU-7000-EG-530 UR radial and/or linear EUS (Fujinon 4400 SU-7000-EG-530 UT) echoendoscopes were used. The solid or cystic nature of the lesion detected with EUS, its size, location, number, morphological characteristics (septation, lobulation, thickened cyst wall, mural nodule, calcification on cyst wall, connection to the pancreatic duct, dilation of the main pancreatic duct), invasion to vascular structures, presence of lymph nodes, and adjacent or surrounding organ metastases were investigated. The morphological characteristics of the cyst, such as the presence of a thickened cyst wall (>3 mm), mural nodule, and main pancreatic duct dilation (caliber >5 mm), were used to distinguish neoplastic cysts from nonneoplastic cysts. The criteria considered for suggesting malignant lymph nodes were a round shape, a short axis diameter >10 mm, and hypoechogenic mass with a distinct border.

#### Endoscopic Ultrasonography-Guided Fine Needle Aspiration

Selection criteria for EUS-FNA: presence of symptoms, increase in cyst size by >3 mm, presence of “worrisome features” such as wall thickness, pancreatic duct dilatation, mural nodule, presence of distal pancreatic atrophy, and contrast enhancement on CT. For the aspiration procedure, a linear EUS (Fujinon 4400 SU-7000-EG-530 UT) device was used. Before the procedure, the thrombocyte count, activated partial thromboplastin time, and prothrombin time were measured. Intravenous antibiotics were administered to the patients 30 minutes before EUS-FNA. Oral antibiotic treatment was maintained for 3 days after the procedure. For the aspiration procedure, a 22G needle was usually used. However, for large and thickened-walled cysts and mucinous cysts, a 19G needle was preferred. Mostly cysts were entered once and aspiration was continued until the cysts disappeared as much as possible. In cases with a mural nodule and thickened wall, after aspiration of fluid, the cyst was entered again, and the cyst wall was punctured at the far wall of the cyst. The pancreatic cyst fluid was separated for cytopathological examination. If more than 1 mL of fluid was aspirated, it was sent to the biochemical laboratory for the determination of carcinoembryonic antigen (CEA), carbohydrate antigen (CA 19-9), and amylase levels.

#### Cytopathological Evaluation

Cytopathological evaluation was performed by a single experienced cytopathologist at Ankara University Faculty of Medicine, Pathology Department. After the material was spread on the slide, it was dried using the air or alcohol fixation technique, and after being stained with May–Grunwald Giemsa or Papanicolaou, it was evaluated.

Cyst fluid and serum were analyzed in the central laboratory of Ankara University Ibn-i Sina Hospital. In the analysis of cyst fluid, amylase was investigated in the Beckman Coulter Unicell DxC 800 device enzymatically, CEA and CA 19-9 in the Beckman Coulter Unicell DxC 800 device by the chemiluminescence test method. The diagnoses made with CT and EUS were compared with those in EUS-FNA cytopathological evaluation reports, which were considered the gold standard. Because it is possible to identify the epithelial features and atypical cells of PCNs by cytopathological evaluation, EUS-FNA was accepted as the gold standard in the definition of neoplastic cysts.

### Statistical Analysis

For patient records and statistical analyses, Statistical Package for the Social Sciences Statistics software, version 16.0 for Windows (SPSS Inc., Chicago, Ill, USA) was used. Descriptive statistical methods were used to determine the characteristics of pancreatic cysts. Continuous data were expressed as mean ± standard deviation and median (minimum–maximum), categorical data as frequency and percentile. For continuous variables, that were not normally distributed, a nonparametric test was used, and a *P*-value of <.05 was considered statistically significant. Sensitivity and specificity values of CT and EUS and 95% CIs of these values were calculated using cytopathology results as the gold standard. The diagnostic capacities of CT and EUS were evaluated and compared using receiver operating characteristic (ROC) curve analysis. In the presence of a significant cutoff value, the sensitivity and specificity of the tests were evaluated.

## Results

The study group included 212 patients. The most common symptom was abdominal pain (52.8%). Patients’ characteristics and symptoms are presented in Table 1.

In our study of 212 patients with pancreatic cysts, only 12 (5.6%) underwent surgical resection.

### Computed Tomography Characteristics of the Pancreatic Cysts

In CT, 142 cases (74.3%) had 1 cyst, 17 (8.9%) had 2 cysts, 7 (3.7%) had 3 cysts, 1 (0.5%) had 4 cysts, and 19 (9.9%) had multiple cysts. The cysts were most commonly located in the head of the pancreas in 85 (45.5%), followed by 48 (25.7%) in the body, and 26 (13.9%) in the tail. The remaining cysts had a multifocal location. The median sizes of neoplastic and nonneoplastic cysts were 19 mm [3 mm-75 mm] and 30 mm [3.5 mm-120 mm], respectively, with no significant difference between the groups (*P* > .05).

According to the morphological diagnosis from CT, 70 (36.5%) cysts were reported to be neoplastic, 41 (21.4%) were nonneoplastic, and 81 (42.2%) were undefined. Forty-three (21.7%) cysts were diagnosed as IPMN, 30 (15.2%) as PC, 12 (6.1%) as MCN, 7 (3.5%) as SCN, 7 (3.5%) as true cyst, and 72 (36.4%) remained undefined. Additionally, in 15 (7.5%) cases, there were at least 2 diagnoses, and 9 (4.5%) cases were classified under the category of the “other group” (pancreatic duct dilation, hematoma, and abscess).

### Endoscopic Ultrasonographic Characteristics of the Pancreatic Cysts

In EUS, 171 cases (81%) had 1 cyst, 25 (11.8%) had 2 cysts, 7 (3.3%) had 3 cysts, 7 (3.3%) had multiple cysts (>5 cysts), and 1 (0.6%) had 5 cysts. The cysts were most commonly located in the head of the pancreas in 98 (46.4%) patients, followed by 64 (30.3%) in the body, and 18 (8.5%) in the tail. The remaining cysts had a multifocal location. The median sizes of neoplastic and nonneoplastic cysts were 20 mm [4 mm-85 mm], and 12 mm [3 mm-140mm], respectively, with a significant difference between groups (*P* = .004).

According to the morphological diagnosis from EUS, more than half of the pancreatic cysts were reported as neoplastic [n: 135 (63.7%)], 56 (26.4%) were identified as nonneoplastic, and 21 (9.9%) remained undefined. Morphologically, 54 (25.6%) cysts were identified as MCN, 62 (29.4%) as IPMN, 31 (14.7%) as true cyst, 21 (10%) as PC, and 9 (4.3%) as SCN, whereas 19 (9%) had 2 different morphological diagnoses. None of the patients had complications due to EUS.

The morphologic characteristics, location, and demographic findings of PCNs subtypes according to EUS and CT are presented in Table 2.

### Cytopathological Characteristics of the Pancreatic Cysts

Out of the 212 cases with pancreatic cysts, 125 (59%) underwent EUS-FNA with a single needle pass in 88 (84.6%), 2 needle passes in 15 (14.4%), and 3 needle asses in 1 (1%) case. None of the patients had complications due to the EUS-FNA procedure.

Out of 125 cases that underwent EUS-FNA, cytopathology reports were available in 121. Out of these 121 cases, 63 (52%) were identified as neoplastic and 29 (24%) were classified as nonneoplastic. In 29 (24%) cases, the specific type could not be identified. Based on cytopathology, 53 (44.1%) cysts were diagnosed as MCL (MCN in 26, IPMN in 14, and differential diagnosis could not be made between MCN and IPMN in 13 cases), 10 (8.3%) as PC, and 5 (6%) as SCN. The remaining cytopathology reports were categorized as nondiagnostic, benign cytology, and others (2 adenocarcinoma, 1 neuroendocrine tumor (NET), 1 duplication cyst). In 1 of the 2 cases diagnosed with adenocarcinoma, CT could not indicate the cyst type, whereas EUS interpreted it as a real cyst, and in the other, both CT and EUS interpreted it as IPMN. In the patient with a diagnosis of NET, the diagnosis was not specified by CT and was interpreted as MCN by EUS. While the CT scan of the patient with a duplication cyst was interpreted as a PC, EUS data could not be obtained.

### Comparison of the Cyst Characteristics Determined by Computed Tomography and Endoscopic Ultrasonography with the Cytopathological Findings

**Figure 1:**
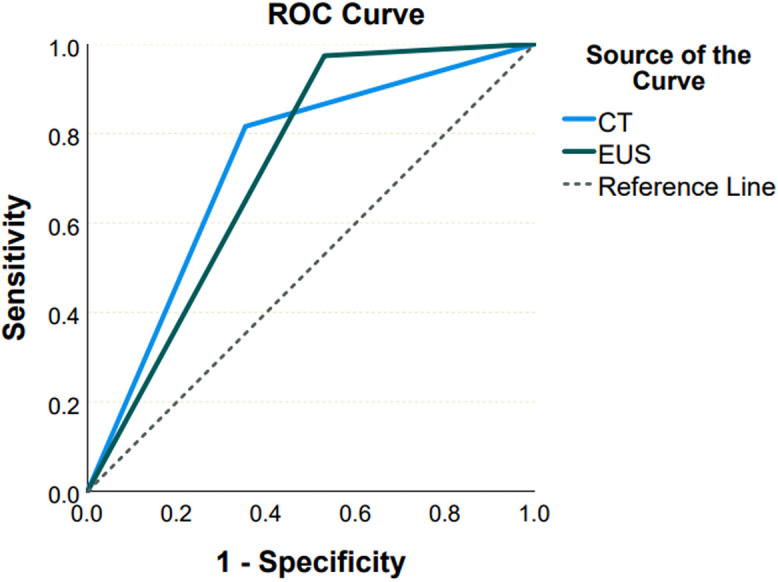
Comparison of receiver operating characteristics curve of CT and EUS. CT, computed tomography; EUS, endoscopic ultrasonography.

Out of the 63 neoplastic cases confirmed by cytopathology, 59 (96.7%) were diagnosed as neoplastic by EUS, whereas among the 29 nonneoplastic cases confirmed by cytopathology, 11 (37.9%) were diagnosed as nonneoplastic by EUS. Among the 63 neoplastic cases, 32 (57.1%) were diagnosed as neoplastic by CT, whereas out of the 29 nonneoplastic cases, 11 (40.7%) were diagnosed as nonneoplastic by CT. The sensitivity, specificity, and diagnostic accuracy of CT and EUS are presented in Table 3. The ROC analysis revealed that CT (area under the curve (AUC): 0.731, 95% CI: 0.599-0.864, *P* = .001) and EUS (AUC: 0.722, 95% CI: 0.597-0.847, *P* < .001) had significant predictive properties for neoplastic cyst diagnosis. The difference in the paired-sample area under the ROC curves was not found to be statistically significant (AUC difference: 0.009, *P* = .899) (Figure 1). Although the sensitivity of EUS was numerically higher than that of CT, it did not achieve statistical significance.

### Laboratory Characteristics of the Pancreatic Cyst Fluid

Amylase, CEA, and CA 19-9 levels of the PCNs and NNPCs are presented in Table 4. There was no statistically significant difference between the median amylase, CEA, and CA 19-9 levels of the neoplastic and nonneoplastic cysts (*P* > .05).

When the cutoff value of CEA was set at ≥192 ng/mL for differentiating MCLs from non-MCLs, its sensitivity was found to be 38.5% with a specificity of 100%. At the same cutoff value, the sensitivity of CEA in differentiating between neoplastic and nonneoplastic cysts was 33.3% with a specificity of 85.7%. When the cutoff value was set at ≥110 ng/mL for CEA, its sensitivity in differentiating MCLs from non-MCLs was found to be 42.3%, with a specificity of 85.7%. At the same cutoff value, the sensitivity of CEA in differentiating between neoplastic and nonneoplastic cysts was 38.9% with a specificity of 85.7%. The CEA levels were significantly different between the MCLs (median: 10.4 ng/mL, mean: 1168 ng/mL) and non-MCLs (median: 2 ng/mL, mean: 3 ng/mL) (*P* < .05).

In our study, when amylase level ≤250 uI/L was accepted as cutoff value, in the differentiation of MCNs from PC, the sensitivity of amylase was determined to be 50% with a specificity of 100%, and IPMNs from PC, the sensitivity was 44.4% with a specificity of 100%.

## Discussion

Our research findings underscore the efficacy of EUS and CT as valuable techniques in differentiating neoplastic cysts from nonneoplastic cysts in comparison with EUS-FNA as the gold standard. When EUS and CT were compared, the sensitivity of EUS appeared numerically superior to that of CT. Although this disparity did not reach statistical significance, it remains noteworthy in the context of clinical practice, suggesting a potentially meaningful trend for practitioners. In previous studies, 85% of pancreatic cysts were reported as PCs and 15% were identified as neoplastic cysts.^12,13^ In our study, neoplastic cysts were more common. The risk of selection bias should be taken into consideration for this result; thus, in this study, we included only patients referred to the gastroenterology department for EUS. In recent studies based on cytopathological evaluation, 60% of pancreatic cysts were reported as MCLs.^14-16^ In our study, the most common subtype of pancreatic cysts was MCL, accounting for 53 (44.1%) cases. The fact that the non-diagnostic group rate was higher than expected may explain the lower MCL rate in our study compared with the literature.

In the literature, as in our study, most of the cases were symptomatic, and the most common symptom was abdominal pain.^12,17^ In our study, congruent with previous studies, female patients accounted for more than male patients, and pancreatic cysts were detected most commonly in the head of the pancreas with CT and EUS.^9,12,17-19^

In our study, according to EUS results, the sizes of neoplastic cysts were significantly larger than those of nonneoplastic cysts. In the literature, the relationship between cyst size and malignancy was evaluated, and the risk of malignancy was associated with larger cysts. The rate of malignancy was 15% when the cyst size was 3-5 cm, and the risk of malignancy was over 30%.^20^ When the cyst size was >5 cm. Although, there is a strong correlation between cyst size and the risk of malignancy, no specific cutoff value for cyst size has been conclusively determined to reliably predict the risk of malignancy. Notably, for asymptomatic lesions smaller than 2 cm, the risk of malignancy is considerably low.

Endoscopic ultrasonography had significantly higher sensitivity than CT, and EUS was superior to CT in demonstrating septation, mural nodules, thickened wall, connection to the pancreatic duct, and dilation of the pancreatic duct. There are other techniques that have been recently used such as contrast-enhanced EUS (CE-EUS) and a specialized contrast-harmonic mode, referred to as CH-EUS. Multiple studies have shown the utility and value of CE-EUS in determining features of mural nodules. The CH-EUS demonstrated enhanced diagnostic accuracy in detecting and characterizing malignant mural nodules.^21^

Diagnostic accuracy of CT in differentiating between neoplastic and nonneoplastic cysts was reported as 39%-44.7%,^10^ suggesting that the misinterpretation rate of CT in neoplastic cysts was very high. Diagnostic accuracy of EUS in differentiating neoplastic cysts from nonneoplastic cysts was 70.4%.^10^ In our study, diagnostic accuracy of CT and EUS was found to be higher than that reported in the literature. When interpreting these findings, the risk of referral bias should be taken into consideration, since all patients included in our study were all specifically referred to the gastroenterology department for EUS. Diagnostic accuracy of CT and EUS in differentiating MCLs from non-MCLs varied in the literature (44%-94%).^22,23^ In our study, diagnostic accuracy of EUS was found to be higher than that of CT in both neoplastic cyst and mucinous cyst differentiation.

In addition to cytopathology, the biochemical findings and tumor markers (amylase, CEA, and CA 19-9) of the pancreatic cyst fluid are also assessed to make a diagnosis. In our study, amylase, CEA, and CA 19-9 levels were evaluated in the cyst fluid. Although there was a numerical difference in the mean values of amylase, CEA, and CA 19-9 in cyst fluid between neoplastic and nonneoplastic cysts, there was no statistical difference, which might be attributed to the small sample size. Furthermore, the diversity observed in the mean and median values of CEA in nonneoplastic cysts, which were found to be higher than those reported in the literature, could be attributed to several factors. Primarily, our study’s adoption of cytology as the gold standard might have influenced these results. It is noteworthy that the limited number of patients undergoing resection could have impacted the analysis. Specifically, cases not subjected to resection were predominantly evaluated as benign based on cytopathological assessment. This could have contributed to these variations in CEA values between groups compared with those reported in the literature. Although malignant neoplasms tend to have higher CEA levels, no direct and conclusive correlation between CEA levels and the presence of malignancy has been reported in the literature.^9,24^ However, because of the wide range of sensitivity and specificity of chemical analyses, it is difficult to make an interpretation.^7,25^ The sensitivity of CEA was 63% with a specificity of 93% in differentiating between IPMNs and MCNs.^26^ Different cutoff values for CEA were evaluated in the studies. In 2 different studies, when the cutoff value of CEA was set at <5 ng/mL for differentiating MCLs from non-MCLs, its sensitivity was found to be 100% with a specificity of 86%.^9,25^ Van der Waaij et al^27^ reported that cysts with CEA <5 ng/mL were reported as SCNs or PCs with the sensitivity of 50%, a specificity of 95%, and diagnostic accuracy of 67%. When the cutoff value of CEA was set at >800 ng/mL for differentiating MCNs and MCAC (mucinous cystic adenocarcinoma) from SCNs and PCs, its sensitivity was found to be 48% with a specificity of 98%, and diagnostic accuracy of 79%. Sharma et al^28^ reported that when the cutoff value of CEA was set at < 45 ng/mL for differentiating MCLs from non-MCLs, its sensitivity was found to be 88.5% with a specificity of 96.8%. Okasha et al^29^ reported that when the cutoff value of CEA was set at 105 ng/dL for differentiating MCLs from non-MCLs, the sensitivity was found to be 80% with a specificity of 77%. The most common cutoff level is 192 ng/mL for CEA.^9^

In our study, the cutoff value of CEA was set at ≥192 ng/mL for differentiating neoplastic cysts from nonneoplastic cysts, and its sensitivity was found to be 33.3% with a specificity of 85.7%, indicating that CEA is not conclusively reliable for the differentiation of neoplastic cysts. However, at the same cutoff level for CEA for differentiating MCLs from non-MCLs, its sensitivity was found to be 38.5% with a specificity of 100% in. In our study, the sensitivity of CEA in differentiating MCLs from non-MCLs was observed to be lower than that reported in the literature. This diversity might be attributed to several factors such as the retrospective nature of the study, a relatively small sample size, and the presence of missing data during the analysis. Similar to the literature, in our study, a high specificity rate was found, and the high-level of CEA was considered to be related to the mucinous nature of the cyst.^7,9,24^

In PCs and main duct IPMNs, the amylase level in the cyst fluid tends to be notably elevated. Typically, these levels reach thousands and are rarely <250 U/L in PCs. Conversely, in SCNs, the amylase level is significantly low.^27^ In a study, it was reported that cysts with amylase level <250 U/L were more likely to be SCNs, MCNs, or MCACs with a sensitivity of 44%, and a specificity of 98%. Hence, it can be inferred that PCs were largely excluded based on this criterion.^27^ In our study, the findings regarding amylase levels were congruent with the literature; there were no PCs with the amylase level <250 uI/L. Although amylase is a helpful marker in lower levels, according to Ngamruengphong and Lennon,^30^ it was not considered to contribute to the differential diagnosis in higher levels because high amylase levels did not differentiate IPMNs from MCNs. The utility of measuring amylase levels in diagnosing pancreatic cystic lesions has been discredited. This is because while amylase can differentiate PCs from nonmucinous cysts, it lacks the ability to differentiate between mucinous cysts and nonmucinous cysts.^31^ The European Study Group on Cystic Tumours of the Pancreas^23^ reported a combined analysis of CEA, lipase levels, and cytology in cyst fluid related to better accuracy rates for differentiating mucinous from nonmucinous PCNs. Recently, there have been further studies analyzing DNA, RNA, and other molecular markers in cyst fluid to make a more decisive evaluation; however, these tests have not yet been established in clinical practice, and in our study, these markers were not tested.^32^

This study has several limitations, including its retrospective design, relatively small sample size, reliance on cytopathology as the gold standard, and the inclusion of cases that were cytopathologically non-diagnostic.

Cytopathology was adopted as the gold standard in our study due to the relatively low percentage of patients, specifically 7.4% (n = 12) out of the total 212, who underwent surgical resection. Further studies, evaluating the histopathological findings from a larger cohort of patients undergoing surgical resections rather than relying solely on cytopathology, would be enlightening.

Insufficient material obtained during cyst aspiration, or encountering acellular cyst fluid, despite adequate aspiration material, may explain the presence of non-diagnostic cases upon cytopathological evaluation. The identification of cuboidal epithelium for serous cysts and columnar epithelium for mucinous cysts is essential for differential diagnosis. However, the inability to differentiate between these epithelial types significantly contributed to the classification of cases as non-diagnostic.

Recent studies have determined that the success of histopathological evaluation has increased by a technique referred to as endoscopic ultrasound through-the-needle biopsy. It is a viable technique capable of yielding a high rate of satisfactory specimens for histological examination. A meta-analysis showed that a specific histotype diagnosis could be reliably established in approximately two-thirds of patients. Although the incidence of adverse events was shown to be slightly elevated compared with standard EUS-FNA, severe complications were rare.^33^

The strengths of the study lie in the consistency of the EUS procedure, conducted by a single experienced endoscopist, and the cytopathological evaluations performed by a skilled cytopathologist. This lends credibility to our findings. Additionally, our study holds significance as it provides real-life data derived from a sufficient number of cases within a single center, enhancing its applicability and relevance to clinical practice.

In conclusion, our study significantly contributes to understanding the diagnostic landscape of pancreatic cystic lesions, emphasizing the role of modalities such as EUS and CT in differentiating between neoplastic and nonneoplastic cysts. It is noteworthy for clinical consideration that EUS displayed a higher sensitivity than CT, although the difference was not statistically significant. Distinguishing between MCLs and non-MCLs remains a challenge, with our findings favoring EUS over CT for better accuracy. Further larger cohort studies that integrate evolving diagnostic modalities are warranted to advance accurate and effective diagnostic strategies in clinical practice.

## Figures and Tables

**Table 1.** Patient Characteristics and Symptoms

	n (%)
Age (median years) Total Female Male	62 (10-87) 62 (10-87) 62 (14-84)
Sex Female Male	118 (55.7) 94 (44.3)
Presence of symptoms Yes No Unknown	120 (56.6) 79 (37.3) 13 (6.1)
Symptoms* Abdominal pain Weight loss Vomiting Jaundice Acute pancreatitis Chronic pancreatitis	112 (52.8) 10 (4.7) 8 (3.8) 7 (3.3) 33 (15.6) 9 (4.2)

Data are shown as numbers and percentages.

*Patients had at least one of these symptoms.

**Table 2.** Characteristics of the Pancreatic Cysts at Computed Tomography and Endoscopic Ultrasonography

	IPMN	MCN	MCL	SCN
CT (N) EUS (N)	43 62	12 54	55 116	7 9
Age (median)	65	64.5	65	69.5
Gender	F = M	F = M	F > M	F > M
Location	CT: head and body > tail EUS: body > head > tail	CT: head > body > tail EUS: body > head > tail	CT: head > tail > body EUS: head > tail > body	CT: head > body > tail EUS: head = body > tail
CT features Connection to pancreatic duct Pancreatic duct dilation Lobulation Septation Mural nodule Calcification	9 (64.3%) 4 (28.6%) 4 (28.6%) 2 (14.3%) 1 (7.1%) None	7 (31.8%) 4 (17.4%) 5 (22.7%) 6 (27.3%) None 1 (4.5%)	4 (33.3%) 2 (16.7%) 5 (41.7%) 5 (41.7%) 1 (8.3%) 1 (8.3%)	None None 5 (83.3%) 3 (50%) None 2 (33.3%)
EUS features Connection to pancreatic duct Pancreatic duct dilation Septation Lobulation Mural nodule Thickened wall Calcification	13 (92.9%) 7 (50%) 8 (57.1%) 2 (14.3%) 5 (35.7%) 1 (7.1%) None	7 (26.9%) 6 (23.1%) 12 (46.2%) 6 (23.1%) 7 (26.9%) 7 (26.9%) 1 (3.8%)	3 (25%) 3 (23.1%) 7 (53.8%) 3 (23.1%) 2 (15.4%) 2 (15.4%) None	None None 3 (50%) 4 (66.7%) None None 1 (16.7%)
Postoperative pathology (N)	1	4	5	2

CT, computed tomography; EUS, endoscopic ultrasonography; F, female; IPMN, intraductal papillary mucinous neoplasms; M, Male; MCL, mucinous cystic lesions; MCN, mucinous cystic neoplasms; SCN, serous cystic neoplasms.

**Table 3.** Diagnostic Efficacy of Computed Tomography and Endoscopic Ultrasonography in Differentiating Neoplastic Cysts from Nonneoplastic Cysts and Mucinous Cysts from Nonmucinous Cysts

	CT			EUS		
	Sensitivity	Specificity	Diagnostic Accuracy	Sensitivity	Specificity	Diagnostic Accuracy
N/NNPC (CI)	82.1% (68.2%-91.9%)	61.1% (38.2%-81%)	75.4%	96.7% (90.2%-99.4%)	45.8% (27.1%-65.4%)	82.3%
MCL/NMCL (CI)	95.8% (82.9%-99.8%)	33.3% (6.5%-71.9%)	83.3%	97.7% (90.4%-99.9%)	60% (19.9%-91.9%)	93.8%

CT, computed tomography; EUS, endoscopic ultrasonography; MCL/NMCL, mucinous cyst/non mucinous cyst lesion; N/NNPC, neoplastic/nonneoplastic pancreatic cyst.

**Table 4.** Biochemical Characteristics of the Neoplastic and Nonneoplastic Cysts

	Neoplastic Cyst	Nonneoplastic Cyst	*P*
CEA n Mean Median Range	36 596.2 ng/mL 36.8 ng/mL 0.1-12278 ng/mL	13 42.4 ng/mL 9.1 ng/mL 0.13-304.6 ng/mL	>.05
CA 19-9 n Mean Median Range	37 14333.6 U/mL 1467.8 U/mL 0.8-380000 U/mL	13 2513.9 U/mL 1119 U/mL 3.3-19777.5 U/mL	>.05
Amylase n Mean Median Range	38 43051 U/L 336 U/L 1-707980 U/L	14 265674.3 U/L 12278 U/L 59-2250512 U/L	>.05

## Data Availability

The data that support the findings of this study are available on request from the corresponding author.
